# An Unusual Presentation of Adenoid Cystic Carcinoma

**DOI:** 10.1155/2015/826436

**Published:** 2015-12-24

**Authors:** Kurren S. Gill, Mark A. Frattali

**Affiliations:** ^1^The Commonwealth Medical College, 525 Pine Street, Scranton, PA 18509, USA; ^2^Delta Medix Ear, Nose & Throat, PC, 940 Jefferson Avenue, Scranton, PA 18510, USA

## Abstract

Adenoid cystic carcinoma (ACC) is a relatively rare tumor of epithelial cell origin, most commonly arising from major salivary glands. It is uncommonly found outside the major or minor salivary glands and is especially rare when located in the nasal cavity. Diagnosis and treatment of ACC pose numerous challenges, partly due to its biological behavior of slow growth, high tendency of local recurrence, and perineural invasion. We present the case of a 67-year-old male with complaints of facial pain and swelling, with a CT scan showing a soft tissue mass extending from the right nasal cavity with osseous destruction. Biopsy revealed ACC with perineural invasion. ACC of the nasal cavity continues to pose diagnostic and therapeutic challenges to physicians. Because this rare pathology presents in a vague manner, early diagnosis requires a high index of suspicion for this disease and close follow-up care. Since ACC of the nasal cavity is seldom reported in the literature, it is our hope that reporting these rare instances as case reports will heighten physician awareness of this rare disease, allowing for early diagnosis and treatment.

## 1. Introduction

ACC is a rare tumor of epithelial cell origin, comprising 1% of malignant tumors of the head and neck region [1]. The most common symptom is a slow growing mass followed by pain, and it has a relentless course with usually a fatal outcome. It most commonly arises from major or minor salivary glands, contributing to 10% of salivary gland tumors [2]. It is rarely seen in the nasal cavity, wherein the most common site is the lateral nasal wall. In these instances, ACC arises from respiratory epithelium, also known as pseudostratified columnar epithelium, rather than the glandular tissue of salivary glands. Diagnosis and treatment of ACC pose numerous challenges, partly due to its biological behavior of slow growth, high tendency of local recurrence, and perineural spread [3]. ACC tends to spread locally through bony destruction and perineural and perivascular invasion. It is characterized by high rates of local recurrence and distance metastasis, occurring as late as 10 to 20 years following initial treatment [4]. Three basic growth patterns have been identified, tubular, cribriform, and solid, with the cribriform pattern being the most frequent. Most patterns are often mixed, and the solid component of the tumor correlates with aggressive behavior [5]. In this paper, we describe a case of ACC of the nasal cavity, present the clinical and radiologic features, and discuss treatment options, with a thorough review of the world literature.

## 2. Case Report

A 67-year-old male presented to our office with bilateral sinus symptoms beginning 3 months prior. The patient complained of facial pain and tenderness, nasal congestion and obstruction, and sinus pain and pressure. He reported a history of allergies and was a former cigarette smoker for 3 years. Physical exam via nasal endoscopy revealed unilateral polyposis, septal deviation, mucosal edema, and facial deformity of the right nasolabial fold. Neck and orbital examinations were negative, and palpation of the face revealed a firm subcutaneous mass in the right nasolabial fold.

**Figure 1:**
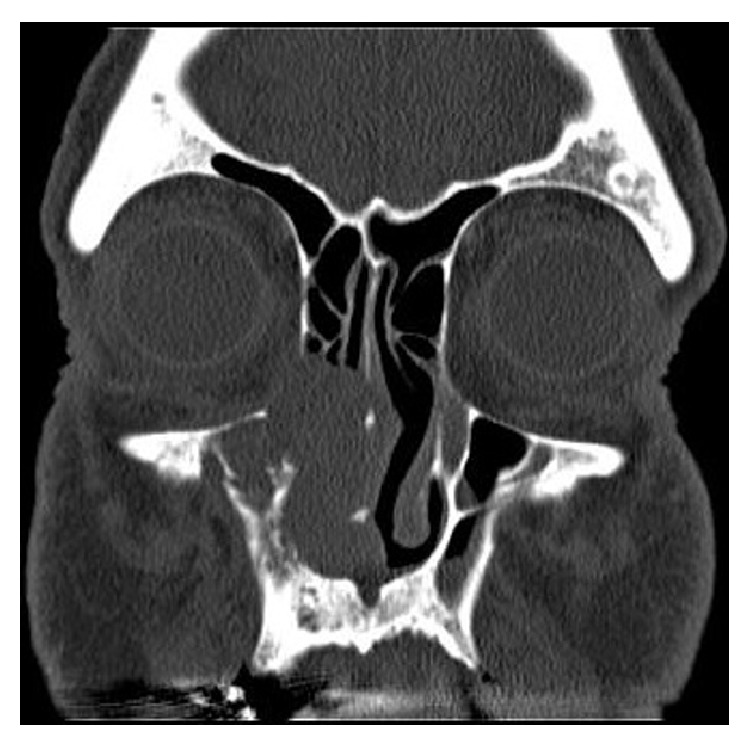
Coronal CT of the sinuses reveals right nasal maxillary mass with medial orbital wall destruction.

CT scan of the sinuses revealed right pansinusitis with a mass originating from the nasal cavity, extending to the right maxillary sinus, and involving the soft tissue of the lateral nasal fold; bone destruction was noted (Figure 1).

**Figure 2:**
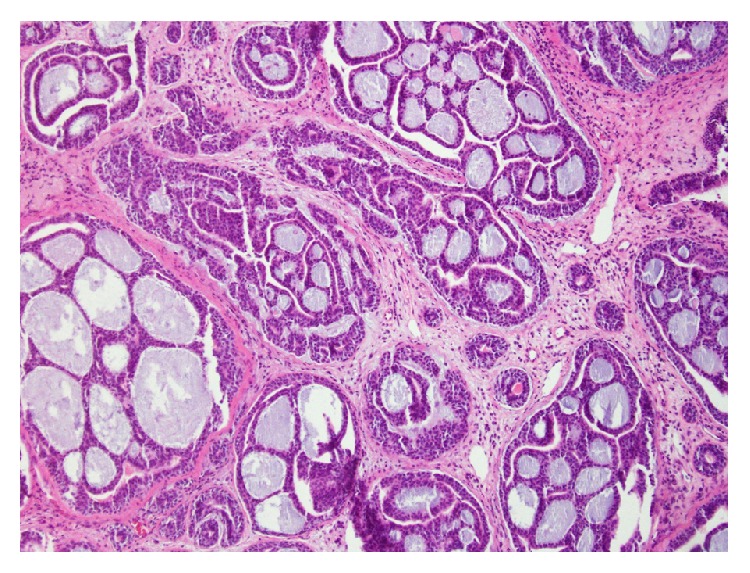
ACC cribriform pattern, characterized by nests of cells with mucopolysaccharide-filled spaces.

The patient then underwent endoscopic nasal polypectomy and biopsies, inferior turbinectomy, total ethmoidectomy, and maxillary sinus antrostomy one week after his initial office visit. Endoscopy revealed a papilliform mass occupying the right nasal cavity, involving the right inferior turbinate and lateral nasal wall with bone destruction. Antrostomy revealed extension of the mass into the maxillary sinus inferolaterally and superiorly. Biopsy of the right inferior turbinate and maxillary sinus revealed soft tissue fragments of ACC (Figure 2).

A follow-up MRI of the orbit/face/neck with and without contrast revealed a residual mass seen along the right nasolabial fold measuring 2.6 × 1.9 cm. The mass extended from the right nare to the external aspect of the right nose, anteriorly to the anterior wall of the right maxillary sinus, medially to involve the nasal septum, and laterally.

A staging PET/CT was conducted and revealed hypermetabolic activity localized to the nasal cavity on the right, additional increased activity localized to the right maxillary sinus, and an extraconal soft tissue density at the inferior aspect of the right orbit and along the infraorbital nerve to the pterygopalatine fossa suspicious for persistent disease. Preoperative staging of the ACC was reported as stage IVA (T4N0M0).

**Figure 3:**
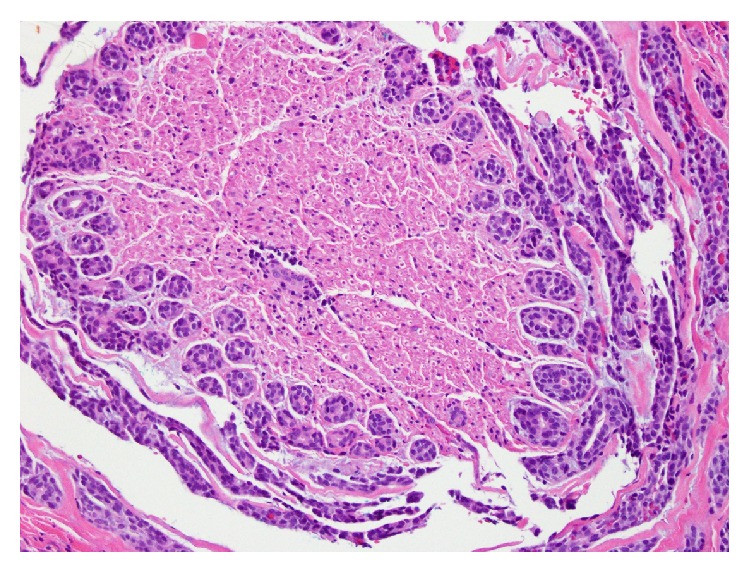
ACC with extensive invasion of the right infraorbital nerve.

Definitive resection was planned and the patient underwent right subtotal maxillectomy with split-thickness skin graft reconstruction and partial palatectomy. Multiple areas were reported to be positive for ACC on frozen section biopsies; of significance was a positive biopsy of the right infraorbital nerve at the apex of the orbital cone exiting the infraorbital fissure (Figure 3).

**Figure 4:**
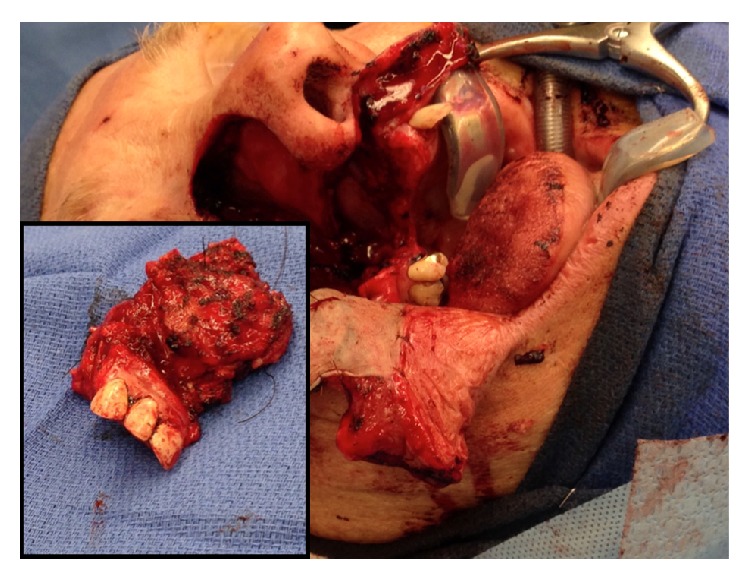
Split thickness graft reconstruction with inset showing resected tumor mass measuring 3.5 cm.

Histological subtype of the ACC was classified as cribriform. Orbital periosteal margins were negative, and thus the right eye was salvaged. There was extensive bone involvement and perineural invasion. The largest gross focus measured 3.5 cm (Figure 4), and the tumor was graded as intermediate G2 moderately differentiated ACC. The patient was discharged 4 days later with plans for postoperative radiation therapy and obturator fabrication and rehabilitation. The patient continues to exhibit no evidence of disease 16 months postoperatively with his obturator in place and maintains functionality of speech and swallowing.

## 3. Discussion

ACC is a relatively rare tumor of epithelial cell origin, most commonly arising from major or minor salivary glands, and comprises 3 to 5% of all head and neck malignancies [6]. The peak incidence is from the fourth decade to the sixth decade, occurring slightly more in women [3]. It usually presents as a slowly growing, firm mass, and patients most commonly report a constant, low-grade dull ache that gradually worsens in severity. Pain as a symptom occurs early in the course of the disease, preceding any significant swelling, likely due to the tumor's predilection for perineural invasion. Presence of lymphadenopathy is uncommon since ACCs do not usually spread to regional lymph nodes. Distant metastasis can occur, with the lung being the most common site [7]. One meta-analysis by Amit et al. found distant metastasis to occur in 29.1% of patients with ACC of the paranasal sinuses and skull base [6]. In addition, extensive bony invasion may be present prior to any radiographic evidence of osseous destruction, which represents another challenge to early diagnosis [8].

Unlike ACC of the salivary glands, ACC can uncommonly occur in other areas of the head and neck region, notably in the nasal cavity. When located outside the salivary glands, ACC can present as nasal congestion or obstruction, facial pain and swelling, and sinus pressure. This contrasts the presentation of ACC in the salivary glands, which presents most commonly as a painless mass or swelling of the parotid, submandibular, or sublingual glands with or without involvement of the facial nerve [9].

ACC of the head and neck, and specifically of the nasal cavity and paranasal sinuses, poses numerous treatment challenges for several reasons: it has a high propensity for local invasion to adjacent structures, making resection more difficult; it is commonly diagnosed late due to its insidious growth; and in 50% of cases it has already exhibited perineural spread at the time of diagnosis [6]. Treatment approach for advanced ACC appears to be best achieved with combined surgical resection and radiotherapy. This was corroborated by Zhang et al.'s retrospective study of 88 patients with ACC in the nasal cavity and paranasal sinuses looking at 5-year and 10-year survival rates. The 5-year and 10-year survival rates of 76% and 41% were obtained in patients who received surgery combined with radiotherapy. The 5-year and 10-year survival rates were 75% and 37% for those treated by surgery alone and 29% and 14% for those treated by radiotherapy alone [10]. Another retrospective study by Liu et al. of 42 patients with ACC of the nasal cavity concluded that surgery combined with high-dose postoperative radiation improves the local control and survival in patients with positive margins, no sufficient margins, or advanced disease [11]. However, multiple other studies by Ellington et al. have reported little to no clear benefit of adjuvant radiotherapy and report the mainstay treatment to remain solely as surgical resection at the present time [12].

Furthermore, ACC is associated with high rates of distant metastasis, which has been noted to occur as late as 10 years after the diagnosis of the primary lesion [13]. The overall 5-year, 10-year, and 15-year survival estimates for all stages among head and neck ACC patients were 90%, 80%, and 69%, respectively, according to data from the American Cancer Society based on 1973–2007 surveillance of 3,026 cases of head and neck ACC [13]. In contrast, the 5-year overall survival and disease-specific survival of ACC of the nasal cavity and paranasal sinuses are 62% and 67%, respectively [6], underscoring the poorer prognosis associated with rarer locations of ACC. The debate of whether perineural invasion confers a poorer prognosis for ACC of the paranasal sinuses remains controversial. One meta-analysis of ACC of the nasal cavity by Amit et al. reports that perineural spread had no effect on overall survival (*p* = 0.3) or disease-specific survival (*p* = 0.37) [6]. Furthermore, the histological subtype also confers prognostic value. According to Szanto et al., ACC is graded as follows: cribriform or tubular (grade 1), less than 30% solid (grade 2), or greater than 30% solid (grade 3), with grade 3 representing the worst prognosis [14]. No differences in the rate of distant metastases and overall survival were found between tubular or cribriform, yet cribriform demonstrated a significantly worse prognosis for local recurrence. The solid pattern tumors have the worst prognosis in terms of distant metastases and long-term survival [15]. The histological subtype in our patient was classified as cribriform.

Immunohistochemical staining for biomarkers in ACC can augment current information regarding prognosis. Since c-KIT mutation expression in ACC can range anywhere from 78% to 100%, there has recently been an increasing intrigue in determining the efficacy of c-KIT inhibitors in treatment of ACC [12]. Histological studies have shown that overexpression of c-KIT mutations in tubular and solid ACC subtypes confers a poorer prognosis [15]. Additionally, Ramer et al. found p63 positivity in ACC to be an independent predictor of survival (*p* = 0.012) and it is useful in the distinction of ACC from basaloid squamous cell carcinoma and high-grade neuroendocrine carcinoma [16, 17].

ACC of the nasal cavity as a rare clinical entity continues to pose diagnostic and therapeutic challenges to physicians. This rare pathology presents in a nondescript manner, as facial pain and swelling or sinus congestion and pressure, and early diagnosis requires vigilance for suspicion of this disease and close follow-up. Debates on the most effective treatment modality continue to surface, with studies corroborating and refuting the benefit of adjuvant radiotherapy after surgical resection. We hope that future studies will provide an answer to this question and elucidate the possible role of chemotherapy and c-KIT inhibitors if one exists [15]. Distant metastasis several years following complete surgical resection and a disease-free interval is not uncommon for ACC, and this warrants close follow-up care of these patients in the long-term setting.
